# The Impact of mHealth-Based Continuous Care on Disease Knowledge, Treatment Compliance, and Serum Uric Acid Levels in Chinese Patients With Gout: Randomized Controlled Trial

**DOI:** 10.2196/47012

**Published:** 2024-04-11

**Authors:** Ying Wang, Yanling Chen, Yuqing Song, Hong Chen, Xin Guo, Ling Ma, Huan Liu

**Affiliations:** 1 Department of Rheumatology and Immunology, West China Hospital, West China School of Nursing, Sichuan University, Chengdu, Sichuan, China; 2 School of Nursing, Chengdu University of Traditional Chinese Medicine, Chengdu, Sichuan, China; 3 West China Hospital, West China School of Nursing, Sichuan University, Chengdu, Sichuan, China; 4 Department of Rheumatology and Immunology, West China Hospital, Sichuan University, Chengdu, Sichuan, China

**Keywords:** mHealth, continuous care, gout, treat-to-target, uric acid, Chinese, awareness, management, intervention, disease management, compliance, mobile health

## Abstract

**Background:**

In patients with gout, suboptimal management refers to a lack of disease knowledge, low treatment compliance, and inadequate control of serum uric acid (SUA) levels. Several studies have shown that continuous care is recommended for disease management in patients with gout. However, in China, the continuous care model commonly used for patients with gout requires significant labor and time costs, and its efficiency and coverage remain low. Mobile health (mHealth) may be able to address these issues.

**Objective:**

This study aimed to explore the impact of mHealth-based continuous care on improving gout knowledge and treatment compliance and reducing SUA levels.

**Methods:**

This study was a single-center, single-blind, and parallel-group randomized controlled trial. Participants were recruited at the West China Hospital of Sichuan University in Chengdu, China, between February 2021 and July 2021 and were randomly assigned to the intervention and control groups. The intervention group received continuous care via an mHealth app, which includes modules for health records, 24 weeks of gout-related health education materials, and interactive support. The control group received routine continuous care, including face-to-face health education, paper-based health education materials consistent with the content for the intervention group, and telephone consultations initiated by the patient. Follow-up was conducted at 6 months. Participants’ gout knowledge levels and treatment compliance were measured at baseline and the 12th and 24th weeks, and participants’ SUA levels were measured at baseline and the 24th week. The intention-to-treat principle and a generalized estimating equation model were used to test the effect of the intervention.

**Results:**

Overall, 258 potential participants underwent eligibility assessments, and 120 were recruited and randomized into the intervention (n=60, 50%) and control (n=60, 50%) groups. Of the 120 participants, 93 (77.5%) completed the 24-week study. The 2 groups had no significant differences in sociodemographic or clinical characteristics, and the baseline measurements were comparable (all *P*>.05). Compared with the control group, the intervention group exhibited a significant improvement in gout knowledge levels over time (β=0.617, 95% CI 0.104-1.129; *P*=.02 and β=1.300, 95% CI 0.669-1.931; *P*<.001 at the 12th and 24th weeks, respectively). There was no significant difference in treatment adherence between the 2 groups at the 12th week (β=1.667, 95% CI −3.283 to 6.617; *P*=.51), while a statistical difference was observed at the 24th week (β=6.287, 95% CI 1.357-11.216; *P*=.01). At the 24th week, SUA levels in both the intervention and control groups were below baseline, but there was no significant difference in SUA changes between the 2 groups (*P*=.43).

**Conclusions:**

Continuous care based on the mHealth app improved knowledge levels and treatment compliance among patients with gout. We suggest incorporating this intervention modality into standard continuous care for patients with gout.

## Introduction

Gout is the most common inflammatory arthropathy, mainly affecting men [1]. Over the past few decades, gout has become increasingly prevalent worldwide, and its prevalence and incidence increased by 6.88% and 6.16% in China in 1990 and 2017, respectively [2]. The quality of life of patients with gout is negatively impacted by severe pain and dysfunction resulting from the accumulation of sodium urate crystals in the joints. In addition, the comorbidity [3] and medical burden [4,5] of gout cannot be ignored. Reducing serum uric acid (SUA) levels is known to prevent crystal formation or prompt dissolution. The treat-to-target approach recommends [6] that SUA levels be maintained below 360 μmol/L in all patients with gout. SUA levels should be less than 300 μmol/L in patients with tophi or frequent attacks [6]. Thus, patients with gout should receive continuous urate-lowering therapy (ULT).

Although the treat-to-target approach has been widely recognized, gout control remains suboptimal. In China, patients with gout are mainly managed by rheumatologists. According to a large sample of data from the Chinese Rheumatism Data Center, only 38.20% of Chinese patients with gout met the SUA target in 6 months [7]. In a cross-sectional survey of patients with gout from 14 European countries, 71% reported inadequate gout control [8]. Previous studies have confirmed that improving disease-related knowledge level and compliance with ULT positively influences patients with gout to reach the SUA goal [9,10]. Unfortunately, in patients with gout, disease-related knowledge is not adequate, especially for the duration of ULT [11], target SUA level [11], and drug-related knowledge [12]. Some studies also showed that the compliance with ULT in China was only 9.6% [13]. Therefore, Chinese patients with gout urgently need to improve disease-related knowledge and treatment compliance. ULT is a long-term challenge for patients with gout [6], so professional guidance is required.

Continuous care refers to the ongoing action designed through a system to ensure that patients in the same or different medical care locations can enjoy the same level of collaborative and continuous care [14]. Its main characteristics are continuity, comprehensiveness, coordination, and cooperation. Continuous care is an economical, convenient, and highly acceptable disease management mode [14]. Doherty et al [15] and Miao et al [16] confirmed the benefits of nurse-led continuous care for disease management in patients with gout. In China, the continuous care model [16-18] for patients with gout consists mostly of face-to-face counseling, telephone follow-up, delivering health knowledge manuals to patients, and using social media to send health-related content. Among these modes, face-to-face consultation and telephone follow-up have disadvantages such as low efficiency, poor accessibility, and small coverage; in contrast, health education manuals and the use of social media cannot meet the diverse needs of patients and lack doctor-patient interaction feedback [19], and the low efficiency and insufficient coverage may limit the number of people receiving continuous care. In addition, the quality of existing continuation care programs is uneven [20,21]; only a few studies have described continuous care programs as being based on guideline recommendations [15,17,20] or having rheumatologist involvement [16,21]. Thus, it is necessary to explore new models of continuous care for patients with gout. The “Healthy China 2030” plan [22] outlines the development of internet-based health services and the active exploration of a new model for internet-based, continuous nursing care to provide new ideas for the continuous care of patients with gout.

The Individual and Family Self-Management Theory (IFSMT) identified 3 dimensions of self-management: context, process, and outcomes [23]. It highlights that individual examination of protective and risk factors and patient empowerment can effectively improve short- and long-term patient outcomes. As it can be used to construct self-management programs, we used the IFSMT to develop an app for the continuous care for patients with gout, designing modules (health records, health education, and interactive support) based on the 3 dimensions of the IFSMT, and identified near-term or long-term observation indicators. Therefore, this study aimed to assess the impact of mobile health (mHealth)–based continuous care on improving gout knowledge level and treatment compliance and controlling the SUA level in Chinese patients with gout.

## Methods

### Study Design

This study was a single-center, single-blind, and parallel-group randomized controlled study and complied with the CONSORT (Consolidated Standards of Reporting Trials) guidelines [24] (Checklist 1).

### Participants

Potential participants were recruited using a convenience sampling method from the West China Hospital of Sichuan University in Chengdu, China, between February 2021 and July 2021. The inclusion criteria for the participants were as follows: (1) met the 2015 gout classification criteria modified by the American College of Rheumatology and European Alliance Against Rheumatology [25,26]; (2) aged ≥18 years; (3) knew about their disease status; (4) used Android smartphones independently; (5) was able to read and understand the questionnaire and the content of this intervention; and (6) agreed to voluntarily participate in the study. Patients with cognitive dysfunction or mental illness and those using other gout management apps or participating in other research programs were excluded.

### Settings and Procedures

A total of 3 registered nurses were assigned to implement the program, and they were assisted by 2 rheumatologists. All investigators were fully aware of the study protocol and questionnaires to ensure a complete understanding of the study-related principles and implementation.

Participants were first invited by their attending rheumatologist to join the study. Once verbal consent was obtained, a research nurse approached each participant to provide further study information and obtain written informed consent to complete the baseline data collection. Randomization was then conducted. Another nurse assisted the intervention group participants to scan a special QR code to download and install the Gout Intelligent Management app (Android version 1.0) and complete the registration. The participants used the app for free during the study. In the control group, the participants were given one-to-one health education on the spot and received health education materials and a card marked with a consultation phone number.

### Randomization, Allocation, and Blinding

We used SPSS software (version 25.0, IBM Corp) to generate a 1:1 randomization sequence. The randomization sequences were placed in opaque sealed envelopes to hide the assignment message and were kept by 1 investigator. After the participant signed the informed consent form and completed the baseline information, another investigator opened the envelope and assigned the participant to the intervention or control group.

### Intervention Group

#### Development of the mHealth App for Continuous Care

**Figure 1.**
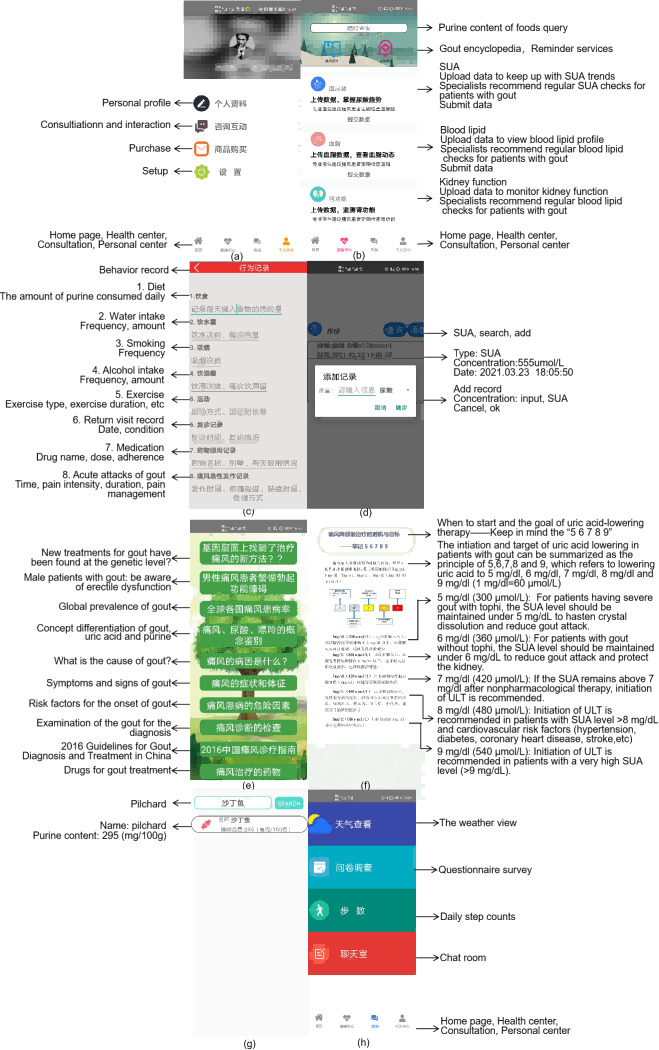
The continuous care modules of the Gout Intelligent Management app: (A-D) health records module, (E-G) health education module, and (H) interactive support module. SUA: serum uric acid; ULT: urate-lowering therapy.

In China, the mHealth support necessary for the self-management of patients with gout includes instrumental support (health education, hospital registration, setting reminders, and shopping), relational support (interactions with health care providers and fellow patients), and psychological support (helping patients mitigate various negative emotions) [27]. Using the IFSMT, we developed the Gout Intelligent Management app, an mHealth app for continuous gout care. The main modules of the app were designed through a comprehensive literature review and expert consultations. The main functions that relate to continuous care are shown in Figure 1. According to the context dimension of the IFSMT, we designed the app’s health records module to integrate demographic data, disease-related information, lifestyle information, and behavior records (activities, SUA, drug administration, drinking water, and diet). Users can register on the app and record their health information by completing a questionnaire. Subsequently, users can log in to view and update their information as needed.

We designed the health education module of this app according to the process dimension of the IFSMT. We referred to an authoritative guide [26,28,29] developed in a previous study on health education [27] and edited it into 24 weeks of health education graphic and text materials (Table 1). We invited 4 rheumatologists, 4 rheumatology nurses, and 2 patients with gout to revise the draft of the health education materials to ensure accuracy and readability. These materials can be shared with users through the app. The gout encyclopedia module in the app presents text and video information, such as basic disease knowledge, treatment, diet, lifestyle, psychosocial management, and new progress in gout treatment, for users to consult independently. In addition, a food purine query module was developed to provide users with common food purine content. Furthermore, we designed an interactive support module for remote consultation, in which users can add the researchers as friends and initiate text, voice, or video sessions, and the researchers can provide a web-based consultation (gout management–related content only). In the clinic coordination function, the app helps users adhere to routine clinical visits. Users can voluntarily participate in group interactions initiated by researchers or other users to share disease-related experiences.

**Table 1.** Content of the 24 weeks of health education materials.

Week	Theme	Content
1	Diet advice	Suggested foods Restricted foods Food to avoid
2	Diet advice	Purine content in common foods Introduction to the purine query module of the Gout Intelligent Management app
3	Diet advice	Establishing good eating habits Recommended intake of energy and nutrients
4	Effects and side effects of medications	The function and precautions of colchicine in the treatment of acute gout attacks The function and precautions of NSAIDs^a^ in the treatment of acute gout attacks The function and precautions of corticoids in the treatment of acute gout attacks
5	Effects and side effects of medications	The function and precautions of allopurinol The function and precautions of febuxostat The function and precautions of benzbromarone The function and precautions of ProSyl
6	Prevention and management of an acute attack	The predisposition to acute attacks of gout The main principles of treatment for acute attacks of gout Medication options for acute attacks of gout Can urate-lowering therapy be used during an acute gout attack?
7	Prevention and management of an acute attack	Improve your lifestyle Treatment to target Pharmacologic prophylaxis as necessary
8	Lifestyle advice	Effects of smoking on gouty arthritis Association between alcohol consumption and gout
9	Lifestyle advice	How to control your weight
10	Lifestyle advice	Exercise mode Exercise intensity Exercise time Exercise frequency Precautions of exercise
11	Urate-lowering therapy	Timing of urate-lowering therapy Target of urate-lowering therapy Course of urate-lowering therapy
12	Basic disease-related knowledge	The etiology of gout Risk factors of gout
13	Basic disease-related knowledge	What is purine? Relationship between purine, serum uric acid, and gout
14	Basic disease-related knowledge	The course of gout Stages of gout and their characteristics
15	Associated comorbidities	Cardiovascular disease Chronic kidney disease
16	Treatment of tophi	How are tophi formed? Characteristics of tophi Hazards of tophi
17	Treatment of tophi	Pharmacological treatment of tophi Nonpharmacological treatment of tophi Principles of tophi management
18	Up-to-date medical news and information related to gout	How to maintain a stable mood?
19	Up-to-date medical news and information related to gout	Common auxiliary tests for gout
20	Up-to-date medical news and information related to gout	The prevalence of gout in different regions The prevalence of gout in different racial groups
21	Up-to-date medical news and information related to gout	Pathogenesis of acute gouty arthritis Pathogenesis of chronic arthritis and its systemic damage
22	Up-to-date medical news and information related to gout	2020 ACR^b^ guidelines for gout treatment: management of acute attacks of gout
23	Up-to-date medical news and information related to gout	2020 ACR guidelines for gout treatment: urate-lowering therapy
24	Up-to-date medical news and information related to gout	2020 ACR guidelines for gout treatment: lifestyle advice and comorbidities

a NSAID: nonsteroidal anti-inflammatory drug.

b ACR: American College of Rheumatology.

#### Implementation of the mHealth App for Continuous Care

The intervention group received 24 weeks of continuous care through the mHealth app. The interventions included collecting health records through the app, gout-related health education, and interactive support.

1. Health records: Each participant in the intervention group was registered on the app. They also filled in sociodemographic, disease, and lifestyle information. Subsequently, they can update this information independently.
2. Health education: We used the app to send web-based health materials to the intervention group patients weekly and to remind them to read them. In addition, they can consult the gout encyclopedia and identify the purine content of food.
3. Interactive support: The researchers or participants may initiate a reminder or consultation regarding gout management.

### Control Group

The control group received routine continuous care, including face-to-face health education, a health education manual consistent with the intervention group’s web-based health materials, and participant-initiated telephone consultations.

### Measurement Indicators

All participants completed the baseline assessment at enrollment. Gout knowledge and compliance were assessed at baseline and the 12th and 24th weeks, and SUA level was measured at baseline and the 24th week. Data from the intervention group were collected through the app, whereas the those from the control group were collected through a web-based platform [30].

#### Sociodemographic Characteristics

Items in the self-report questionnaire included age, gender, race, BMI, waist circumference, marital status, highest education level, employment, living alone or with family, income, gout-related expenses in the past year, and health insurance.

#### Clinical Characteristics and Lifestyle

The clinical characteristics collected included symptoms, duration since diagnosis, comorbidity, a family history of gout, the SUA level of the previous month, whether they are undergoing ULT, and the presence of gout tophi. The lifestyle questionnaire asked about smoking and drinking status.

#### Gout Knowledge Level

The Gout Knowledge Questionnaire (GKQ), developed by Zhang et al [31], was used to assess the participants’ gout knowledge level. The GKQ was evaluated through test questions, including 10 questions covering the basic knowledge of the pathogenesis, clinical manifestations, treatment, and management of gout. With only 1 correct answer for each question, a correct answer scored 1 point, and an incorrect answer scored 0 points, resulting in a total score between 0 and 10 points. The Flesch-Kincaid grade level for this scale was 4.7, and the Flesch reading ease was 81.4% [31].

#### Treatment Compliance

The Chinese Compliance Questionnaire Rheumatology (CCQR) was used to assess the participants’ treatment compliance. This questionnaire was prepared by de Klerk et al [32]. It was translated into a Chinese version by Zhu et al [33] and verified to have a test-retest reliability of 0.994 and a content validity of 0.635. This questionnaire includes 19 items, with 1-4 points corresponding to “totally disagree,” “disagree,” “agree,” and “totally agree,” respectively. The CCQR score is calculated as *(sum of the score of each item – 19) / 0.57*. The total score ranges from 0 to 100 points, and lower scores indicate worse medication compliance. The Cronbach α of the CCQR in this study was 0.870.

#### SUA Level

To measure SUA levels, fasting venous serum was drawn from the participants. Uricase was then used to detect SUA by peroxidase uncoupling.

### Sample Size Estimation

The sample size was estimated using a completely random, 2-sample means sample size formula. The test efficacy was set to significance levels of .90 and .05. According to the randomized controlled trial by Doherty et al [15], the mean SUA difference between the intervention and control groups was 104.64, and the SD of the SUA value was 141.64. Thus, each group required 39 patients. Considering a 20% loss to follow-up, at least 98 participants needed to be included in this study.

### Statistical Analysis

Data analysis was conducted using SPSS (version 25.0, IBM Corp). Demographic, clinical, and lifestyle data between the groups were presented using appropriate descriptive statistics and evaluated for homogeneity using independent 2-tailed *t* tests, Mann-Whitney *χ*^2^ tests, and Fisher exact tests, as appropriate. The changes in GKQ and CCQR scores at the 12th and 24th weeks from baseline between the 2 groups were assessed using generalized estimating equations. The difference in SUA levels between the 2 groups at the 24th week from baseline and comparison between groups at the same time point were assessed using a 2-tailed *t* test for 2 independent samples. The intention-to-treat principle was adopted in the outcome analysis. All participants were included in the generalized estimating equation analysis since all participants provided baseline data. All statistical tests were 2-sided, with the level of significance set at .05.

### Ethical Considerations

This study met the ethical guidelines of the 1975 Declaration of Helsinki. Ethical approval was obtained from the Medical Ethics Committee of West China Hospital in 2020 (ID: 2020898). All participants signed an informed consent form prior to enrollment and were informed that they can withdraw at any time without their medical treatment and rights being affected. Recruited participants will be provided with free disease management services for 6 months and will be assisted by the investigator to seek medical attention or hospitalization in the event of adverse reactions to treatment or other comorbidities during the course of the study. The data collected for this study will be anonymized and used for research purposes only.

## Results

### Participant Recruitment and Retention

**Figure 2.**
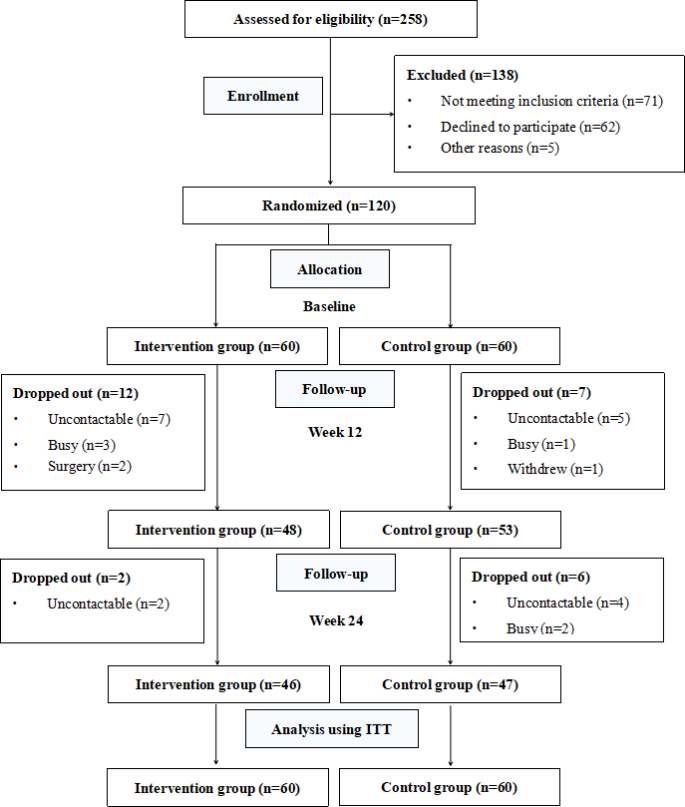
CONSORT (Consolidated Standards of Reporting Trials) diagram for study participant flow and condition allocation. ITT: intention to treat.

Figure 2 shows the recruitment and retention of the participants. Between February and July 2021, a total of 258 potential participants underwent eligibility assessments, and 138 were excluded. We recruited 120 patients with gout and randomized them into the intervention group (n=60, 50%) and the control group (n=60, 50%). Of the 120 participants, 93 (77.5%) completed the 24-week study. Since we used the intention-to-treat principle, we included all 120 patients for statistical analysis.

### Baseline Characteristics of the Participants

The baseline characteristics of the participants are shown in Table 2. The mean age was 41.53 (SD 12.58) years. The average BMI was 26.28 (SD 3.27) kg/m^2^, and the average waist circumference was 93.24 (SD 12.04) cm. We collected 115 participants’ SUA level; the average SUA level was 457.67 (SD 141.83) μmol/L. Of the 120 participants, 119 (99.2%) were male, 116 (96.7%) were of Han ethnicity, 97 (80.8%) were married, and 73 (60.8%) had a college degree or above. Among them, 96 (80%) were receiving ULT, 31 (25.8%) had tophi, 50 (41.7%) had hyperlipidemia, 18 (15%) had hypertension, 15 (12.5%) had kidney disease, and 6 (5%) had diabetes. Furthermore, 41 (34.2%) participants smoked, and 58 (48.3%) drank alcohol. The 2 groups had no significant differences in sociodemographic or clinical characteristics (all *P*>.05).

**Table 2.** Sociodemographic profile of all participants, within and between the intervention group and control group.

Characteristics		Total (n=120)	Intervention group (n=60)	Control group (n=60)	*P* value
Age (y), mean (SD)		41.53 (12.58)	43.67 (12.65)	39.40 (12.24)	.06^a^
BMI (kg/m^2^), mean (SD)		26.28 (3.27)	26.25 (3.55)	26.30 (2.99)	.93^a^
Waist circumference (cm), mean (SD)		93.24 (12.04)	92.08 (11.25)	94.52 (12.84)	.29^a^
SUA^b^ (μmol/L), mean (SD)^c^		457.67 (141.83)	455.13 (140.70)	460.18 (144.11)	.86^a^
**ULT^d^** **, n (%)**					.36^e^
	Yes	96 (80)	46 (77)	50 (83)	
	No	24 (20)	14 (23)	10 (17)	
**Marital status, n (%)**					.25^e^
	Single, divorced, or widowed	23 (19.2)	9 (15)	14 (23)	
	Married	97 (80.8)	51 (85)	46 (77)	
**Educational level, n (%)**					.56^e^
	Junior high school or below	31 (25.8)	15 (25)	16 (27)	
	Senior high school	16 (13.3)	10 (17)	6 (10)	
	College or above	73 (60.8)	35 (58)	38 (63)	
**Monthly per capita income (CN ¥; CN ¥1=US $0.14), n (%)**					.98^e^
	<4000	36 (30)	17 (28)	19 (32)	
	4000-7999	37 (30.8)	19 (32)	18 (30)	
	8000-14,999	23 (19.2)	12 (20)	11 (18)	
	≥15,000	24 (20)	12 (20)	12 (20)	
**Employment status, n (%)**					.81^e^
	Employed	99 (82.5)	50 (83)	49 (82)	
	Unemployed	21 (17.5)	10 (17)	11 (18)	
**Medical insurance, n (%)**					.71^e^
Self-pay		44 (36.7)	23 (38)	21 (35)	
Medical insurance		76 (63.3)	37 (62)	39 (65)	
Symptom duration (mo), median (IQR)		72.00 (35.00-144.00)	72.00 (21.00-131.75)	70.00 (44.00-155.00)	.57^e^
Duration since diagnosis (mo), median (IQR)		58.00 (17.50-131.75)	64.00 (15.25-130.50)	52.50 (17.75-147.50)	.83^e^
Tophi, n (%)		31 (25.8)	17 (28)	14 (23)	.53^e^
**Comorbidity**					
	Hyperlipidemia	50 (41.7)	26 (43)	24 (40)	.71^e^
	Hypertension	18 (15)	11 (18)	7 (12)	.31^e^
	Kidney disease	15 (12.5)	8 (13)	7 (12)	.78^e^
	Diabetes	6 (5)	2 (3)	4 (7)	.34^f^
Smoking, n (%)		41 (34.2)	20 (33)	21 (35)	.85^e^
Drinking, n (%)		58 (48.3)	31 (52)	27 (45)	.47^e^

a Independent samples 2-tailed *t* test.

b SUA: serum uric acid.

c Total: n=115; intervention group: n=58; control group: n=57.

d ULT: urate-lowering therapy.

e Chi-square test.

f Fisher exact test.

### Effects of mHealth App–Based Continuous Care on Outcomes

**Figure 3.**
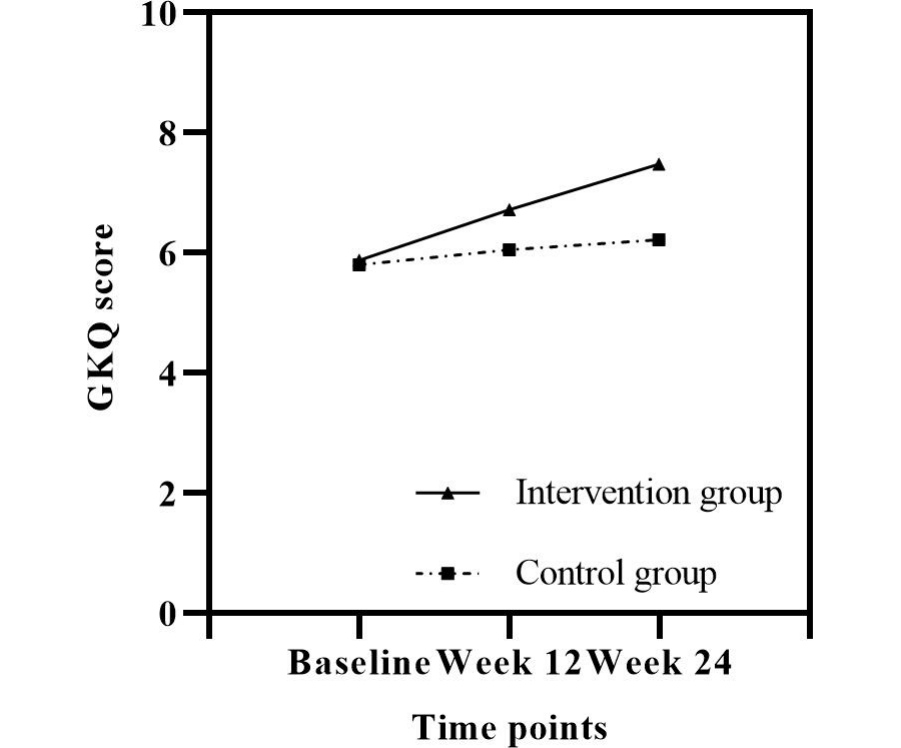
Graphical representations of the mean changes in GKQ scores from baseline to the 24th week. GKQ: Gout Knowledge Questionnaire.

The mean GKQ scores at baseline were 5.88 (SD 2.41) in the intervention group and 5.80 (SD 2.34) in the control group. No significant intergroup differences were observed (*P*=.85; Table 3). At the 12th week, the GKQ scores of the intervention and control groups increased, but this change was not statistically significant (*P*=.06; Table 3). At the 24th week, the GKQ scores of the intervention and control groups continued to increase, and significant differences were observed between the intervention and control groups (*P*<.001; Table 3). Furthermore, a significant interaction term (group × time) of the model at the 12th week (β=0.617, 95% CI 0.104-1.129; *P*=.02) and the 24th week (β=1.300, 95% CI 0.669-1.931; *P*<.001) verified that mHealth-based continuous care effectively increased the GKQ scores over time (Table 4 and Figure 3).

**Table 3.** Intention-to-treat analysis: intervention effect on outcomes.

Outcome and group		Baseline				12th week				24th week			
		Patients, n	Mean (SD)	Difference between groups(95% CI)	*P* value	Patients, n	Mean (SD)	Difference between groups(95% CI)	*P* value	Patients, n	Mean (SD)	Difference between groups(95%CI)	*P* value
**GKQ^a^ score**				0.08 (–0.94 to 0.77)	.85			0.67 (–1.35 to 0.02)	.06			1.27 (–1.93 to –0.61	<.001
	Intervention	60	5.88 (2.41)			48	6.72 (1.88)			46	7.48 (1.69)		
	Control	60	5.80 (2.34)			53	6.05 (1.93)			47	6.22 (1.94)		
**CCQR^b^ score**				0.67 (–4.65 to 6.00)	80			0.99 (–5.62 to 3.64)	.67			5.61 (–10.61 to –6.19)	.03
	Intervention	60	69.80 (14.04)			48	70.82 (11.54)			46	75.00 (12.33)		
	Control	60	70.13 (15.38)			53	69.82 (13.95)			47	69.39 (15.16)		
**SUA^c^ level (μmol/L)**				5.05 (–47.58 to 57.67)	.85			N/A^d^	N/A			15.13 (–22.33 to 52.58)	.43
	Intervention	57	455.13 (140.70)			N/A	N/A			46	396.76 (104.75)		
	Control	58	460.18 (144.11)			N/A	N/A			47	411.88 (97.77)		

a GKQ: Gout Knowledge Questionnaire.

b CCQR: Chinese Compliance Questionnaire Rheumatology.

c SUA: serum uric acid.

d N/A: not applicable.

**Table 4.** Results of the generalized linear model regarding the effects of mobile health–based continuous care on GKQ^a^ and CCQR^b^ scores (n=120).

Outcome and variable			β (95% CI)		SE	*P* value
**GKQ**						
	Group (intervention group vs control group)		0.033 (−0.80 to 0.87)		0.426	.94
	**Time**					
		Baseline	Reference		—^c^	—
		12th week	0.250 (−0.088 to 0.588)		0.173	.15
		24th week	0.417 (0.011 to 0.823)		0.207	.04
	**Group × time**					
		Group × baseline	Reference		—	—
		Group × 12th week	0.617 (0.104 to 1.129)		0.262	.02
		Group × 24th week	1.300 (0.669 to 1.931)		0.322	<.001
**CCQR**						
	Group (intervention group vs control group)		−0.673 (−5.898 to 4.553)		2.666	.80
	**Time**					
		Baseline	Reference		—	—
		12th week	−0.643 (−4.250 to 2.963)		1.840	.73
		24th week	−1.082 (−4.628 to 2.464)		1.809	.55
	**Group × time**					
		Group × baseline	Reference		—	—
		Group × 12th week	1.667 (−3.283 to 6.617)		2.526	.51
		Group × 24th week	6.287 (1.357 to 11.216)		2.515	.01

a GKQ: Gout Knowledge Questionnaire.

b CCQR: Chinese Compliance Questionnaire Rheumatology.

c Not applicable.

**Figure 4.**
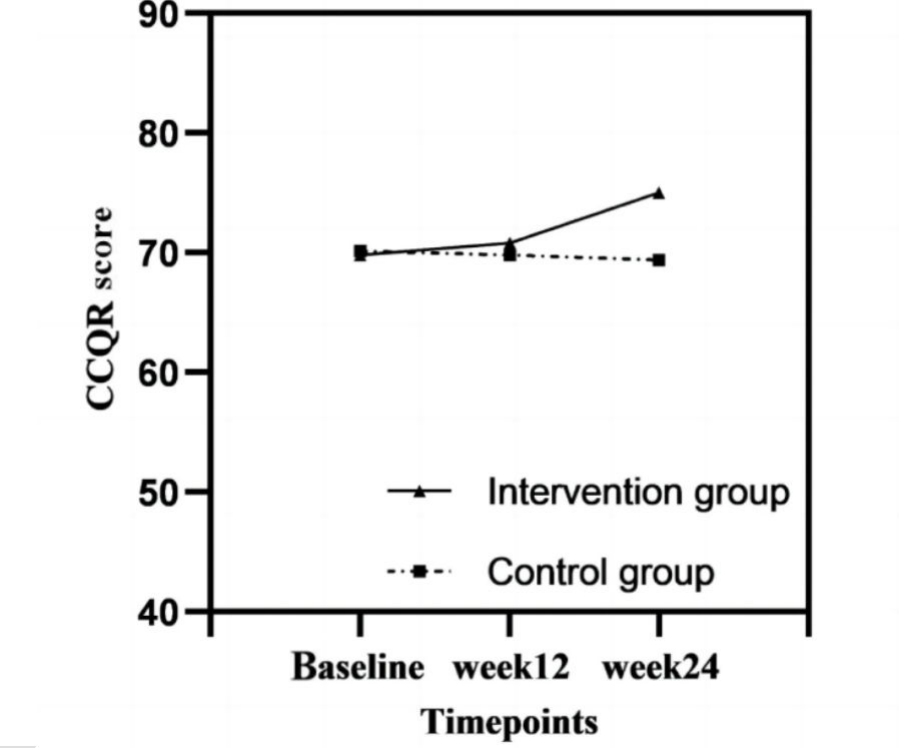
Graphical representations of the mean changes in CCQR scores from baseline to the 24th week. CCQR: Chinese Compliance Questionnaire Rheumatology.

The mean CCQR scores at baseline were 69.80 (SD 14.04) in the intervention group and 70.13 (SD 15.38) in the control group. No significant intergroup differences were observed (*P*=.80; Table 3). The mean CCQR scores gradually increased from baseline to the 24th week in the intervention group and gradually declined in the control group. However, at the 12th week, there was no significant difference between the 2 groups (*P*=.67; Table 3). A significant difference was observed between both groups at the 24th week (*P*=.03; Table 3). Furthermore, no significant interaction term (group × time) of the model was observed at the 12th week (β=1.667, 95% CI −3.283 to 6.617; *P*=.51), but a significant difference was observed at the 24th week (β=6.287, 95% CI 1.357-11.216; *P*=.01), verifying that mHealth-based continuous care effectively increased the CCQR scores over time (Table 4 and Figure 4 ).

The mean SUA levels at baseline were 455.13 (SD 140.70) μmol/L in the intervention group and 460.18 (SD 144.11) μmol/L in the control group. No significant intergroup differences were observed (*P*=.85; Table 3). At the 24th week, the SUA levels in both the intervention and control groups were lower than those at baseline. However, no significant intergroup differences were observed at the 24th week (*P*=.43; Table 3). Furthermore, the decline in SUA levels were compared between the 2 groups. Compared with the baseline data, SUA levels decreased by 71.24 (95% CI −40.38 to 141.12) μmol/L in the intervention group and by 28.50 (95% CI −53.50 to 160.03) μmol/L in the control group. However, the difference between the 2 groups was still not statistically significant (*P*=.55).

## Discussion

### Principal Findings

In this study, the level of gout knowledge refers to patients’ knowledge of gout disease. Similar to the study by Chandratre et al [34], we found that patients with gout had overall low levels of disease knowledge at baseline. In this study, both groups demonstrated an upward trend in GKQ scores over 24 weeks; however, the mHealth app–based continuous care group performed better. Our findings are consistent with those of network-based self-management intervention programs, such as those reported by Oh et al [35], Kang and Lee [36], and Serlachius et al [37]. Additionally, a study in China found that continuous intervention through WeChat can improve gout knowledge [16]. Previous research has shown that patients with gout are more concerned about dietary intervention, gout attack triggers [38], and treatment information [39,40] and enjoy tracking their SUA levels [38]. However, their awareness of epidemiology, comorbidity management, daily lifestyle intervention, and ULT goals is limited [41-43]. Health education is a crucial intervention method for continuous care and a primary means to promote chronic disease self-management. In our previous studies, we identified the health education needs of patients with gout through qualitative interviews [27]. Next, we used this information to develop an mHealth-based gout continuous care program. In addition, compared to traditional continuous care, the app-based intervention provides regular weekly reminders supplemented by a gout encyclopedia, food purine query, web-based consultations, and other auxiliary modules for further patient reinforcement and feedback. This intervention provides patients with knowledge and skills about disease management and may contribute to better treatment adherence and health outcomes. According to Skinner’s [44] reinforcement theory, the impact of reinforcement on individual behavior needs to be achieved through feedback, and individuals adjust, correct, and refine their behavior according to the feedback information. The mHealth app provides multichannel reinforcement and personalized feedback that improve disease-related knowledge in patients with gout. This study shows that continuous care based on a mobile medical app can effectively enhance the knowledge level of patients with gout and prompt continuous gout nursing practices. Medical staff can use the app to provide high-quality health information and improve the knowledge of patients with gout.

In this study, the treatment compliance among patients with gout—the extent to which their medication behavior matched medical orders—had scores of <80 at baseline; this is consistent with the findings of some previous studies [13], meaning that treatment adherence is low in patients with gout. This study showed that continuous care based on an mHealth app could improve treatment adherence in patients with gout. Miao et al [16] reported that a continuous care intervention based on the WeChat public platform could improve treatment adherence in patients with gout, whereas the study by Fields et al [45] failed to improve treatment adherence through a structured gout course and pharmacists’ telephone calls. A qualitative study showed that treatment adherence in patients with gout is influenced by negative experiences and false beliefs [43]. Individuals who obtain information consistent with their health beliefs and behaviors are more likely to adopt recommended health behaviors [23]. The IFSMT proposes that context-specific interventions can reduce risk or promote self-management of conditions through improving knowledge and beliefs [23]. This intervention should address the participants’ needs and strengthen behavior change through various forms of health education, including counseling, communication, peer support, behavioral recording, and reminder services. Therefore, mHealth-based continuous care has advantages over traditional continuous care. Moreover, we also found that the CCQR score was higher in the intervention group at the 12th week than in the control group. However, the difference did not reach statistical significance until the group differences were more pronounced at the 24th week. The transtheoretical model and stages of change propose that human behavior is a gradual and continuous process, requiring creating a supportive environment and self-reinforcement [46]. This study found that mHealth-based continuous care can provide knowledge and skills to support patients with gout, improve treatment compliance, and provide repeated reinforcement, thus playing a more lasting role than traditional continuous care. This suggests that in the practice of continuous gout nursing, medical staff can use mHealth apps to change patients’ incorrect cognition and provide positive incentives and timely communication, enabling patients to master strategies for improved treatment adherence. It also suggests that improving treatment adherence in patients with gout requires a longer intervention period.

This study found that the SUA level for patients with gout at baseline was 457.72 (SD 144.38) µmol/L, which is higher than the treatment target, suggesting that the management of ULT should still be the focus of continuous nursing intervention. Doherty et al [15] reported that patients significantly decreased their SUA levels after a comprehensive intervention, including patient education, patient participation, and ULT. However, the findings of Bulbin et al [20] did not show an improvement in SUA levels through web-based education and reminders. The findings of this study indicate that both intervention strategies contribute to lower SUA levels in patients with gout; however, there were no significant differences between the 2 groups. This study was conducted only for 24 weeks, but the effects of continuous care on SUA levels may be detected in the long term. In addition, another study highlighted that SUA levels were affected by several factors [47]. Therefore, we suggest increasing the follow-up time for further exploration.

### Limitations

This study has several limitations. First, the participants in this study were recruited from 1 tertiary hospital in Sichuan, China, with an inadequate number of female participants, which may limit the generalizability of the study results. Second, the participants in this study need to be able to use smartphones and have certain learning abilities, which may limit the application of this intervention to all patients with gout. Third, the study protocol was planned for 24 weeks, and the sustainability of the intervention’s effects after this period could not be established. Furthermore, this study did not analyze participants’ engagement with the app and its impact on outcome measures.

To address these limitations, the following considerations are essential in future studies: (1) strengthening the links and collaboration between hospitals and communities, in which multicenter studies can further validate the utility of mHealth app–based continuous care; (2) including populations with other rheumatic diseases to expand the scope of mHealth app–based continuous care; (3) communicating with the medical consortium and hospital information systems to solve the difficulty in data collection, which would allow researchers to evaluate the effect of the intervention more comprehensively; and (4) conducting multicenter studies and extending follow-up duration to evaluate the long-term effects of the intervention.

### Implications

The strengths of this study include the development of a nurse-led protocol for continuous care based on patient needs, refined by guidelines, and revised through expert consultations, as well as its representation of the “4C” characteristics of continuous care: comprehensiveness, coordination, continuity, and collaboration. Furthermore, we demonstrated the benefits of intelligent management of mobile medical apps for continuous care for patients with gout.

### Conclusion

We developed an mHealth app for continuous care based on the IFSMT and assessed its effect on disease knowledge, treatment adherence, and SUA levels in Chinese patients with gout. The results show that continuous care based on the mHealth app can improve disease knowledge and treatment adherence in patients with gout. However, the advantages of reducing SUA levels in patients with gout require additional investigation. Our findings suggest that the mHealth app can be used effectively for the continuous care of patients with gout in China.

## Supplementary material

10.2196/47012 Checklist 1 CONSORT-EHEALTH checklist.

## Abbreviations

CCQR: Chinese Compliance Questionnaire Rheumatology
CONSORT: Consolidated Standards of Reporting Trials
GKQ: Gout Knowledge Questionnaire
IFSMT: Individual and Family Self-Management Theory
mHealth: mobile health
SUA: serum uric acid
ULT: urate-lowering therapy
